# Early Canopy Management Practices Differentially Modulate Fruit Set, Fruit Yield, and Berry Composition at Harvest Depending on the Grapevine Cultivar

**DOI:** 10.3390/plants12040733

**Published:** 2023-02-07

**Authors:** Alessandro Mataffo, Pasquale Scognamiglio, Carlo Molinaro, Giandomenico Corrado, Boris Basile

**Affiliations:** Department of Agricultural Sciences, University of Naples Federico II, 80055 Portici, Italy

**Keywords:** *Vitis vinifera*, fruit set, bunch, rachis, canopy, photosynthetic rate, cultivar, ‘Aglianico’, ‘Casavecchia’

## Abstract

The size and number of the berries and the rachis length are the main elements that define bunch compactness in grapevine (*Vitis vinifera* L.). This trait is of scientific and commercial interest because it strongly influences phytosanitary status and quality of the fruits. In this work, we investigated the effect of different canopy management strategies based on apical shoot and/or leaf removal applied at the early stage (pre-bloom) in altering the key determinants of bunch compactness. Specifically, we compared apical defoliation (removal of the first half of the shoot leaves from the top), basal defoliation (removal of the second half), and shoot trimming (removal of the apical half of the shoot) to untreated controls. The work was carried out in two red varieties (‘Aglianico’ and ‘Casavecchia’) that have contrasting bunch compactness (compact and loose, respectively). We measured relevant morphological traits, photosynthetic rates, fertility, fruit set, bunch architecture, and fruit main compositional parameters. This study demonstrates that the position of the removed shoot leaves along with the shoot trimming differentially modified fruit set, the number of berries per bunch, and berry fresh weight and composition at harvest. Nonetheless, the influence on bunch compactness was limited mainly because of photosynthetic and morphological factors strongly associated with the cultivar.

## 1. Introduction

Bunch compactness is an important morphological trait in viticulture because it affects bunch susceptibility to pathogen and hence, fruit quality [1,2,3,4]. A higher degree of compactness couples with micro-climatic conditions that favor fungi development (because of higher humidity in the airspace between berries) and juice leaking from cracked berries, as a consequence of berry-to-berry compression [4]. An insufficient sanitary condition of the grapes also influences harvest, for instance, by urging for an early harvest, and it has therefore a negative impact on wine quality [5,6]. In red wines, for example, the laccase activity of fungi such as *Botrytis fukeliana* could lead to the loss of color and antioxidant activity [6]. Finally, berries in compact bunches are exposed to a more heterogeneous solar radiation, which typically results in a clearly observable heterogeneous maturation within the bunch [7].

Three traits primarily determine bunch compactness: the number of berries per bunch, the berry size, and the length of the rachis and pedicel [4,8]. Different strategies are possible to alter the traits that influence bunch compactness, such as leaf removal, shoot trimming, rootstock selection, and canopy shading [9,10,11,12]. Pre-bloom leaf removal is a canopy management (CM) practice effective in reducing the fruit-set because of temporary carbon limitations caused by leaf removal [13,14,15,16,17]. However, this summer pruning technique, when applied in warm growing areas, can lead to detrimental effects on berry composition [18] mainly due to the excessive exposure of the bunch to solar radiation [19,20]. This could result in an unbalanced sugar-to-acid ratio of berry juice at harvest. Recently, basal defoliation in Cabernet Sauvignon, even at a moderate intensity, is associated with an increase in bunch temperature, which is also present several months after CM [21]. Therefore, it would be interesting to identify alternative pre-bloom pruning strategies suitable to induce the desired carbon limitation during this specific phenological stage but that have, at the same time, a limited impact on bunch microclimate. Shoot trimming is a summer pruning technique that removes leaves only in the apical part of the shoot. In commercial vineyards, shoot trimming is often applied at later phenological stages, from pre-veraison to post-veraison, with the aim of controlling vegetative growth and modulating berry ripening [14,22]. Some authors also reported that late shoot trimming can reduce bunch compactness [3,9,23]. Furthermore, shoot trimming removes shoot apexes together with the leaves. The former, during early canopy developmental stages, represents strong sinks for carbohydrates and significantly competes with flowers during fruit set [15,24,25,26]. An additional alternative to basal defoliation is to remove apical leaves only. This CM allows to decrease the number of leaves without excising the apex and the leaves in the bunch zone. However, the efficacy of pre-bloom apical defoliation in reducing fruit set (and thus bunch compactness) needs to be tested, because it is known that, especially during early stages of canopy development (i.e., between pre-bloom and fruit set), apical leaves have a net CO_2_ exchange rate lower than basal leaves [27]. This may affect the suitability of pre-bloom apical leaf removal as a strategy for reducing bunch compactness, but little investigation has focused on this approach [28]. Another major aspect to be considered is the possible compensative photosynthetic response to leaf removal induced by the different CMs. However, these responses were reported to vary significantly in their intensity and duration [10,17]. Previous studies reported that leaf removal induced an increase in the photosynthetic rate and this resulted in a decrease in the efficacy of CMs [17,29,30], whereas in other cases, this compensation effect was not found [10,14,16,31]. Lastly, it should not be overlooked that key structural traits related to bunch compactness are different among cultivars [8] and therefore, the effect of any CM may vary according to the genetic background.

The aims of this study were to compare the efficacy of three CM practices (basal defoliation, apical defoliation, and shoot trimming) to alter key productive parameters related to bunch compactness, and to evaluate their effect on berry composition at harvest. This work was carried out on two red cultivars characterized by bunches with contrasting compactness, ‘Aglianico’ and ‘Casavecchia’ (having compact and loose bunches, respectively). ‘Aglianico’ is arguably the most important cultivar cultivated in the Campania region, whereas ‘Casavecchia’ is considered an emerging local variety. The results of this study provide insights in the source–sink relationships occurring in grapevines during the phenological stage between pre-bloom and fruit set, establishing a framework for the definition of suitable early canopy management strategies for the manipulation of fruit-set.

## 2. Results

### 2.1. Vine Vegetative Growth

At the phenological stage H, right before the application of the treatments, most of the vegetative characteristics of the shoots selected for the experiment significantly differed between cultivars (Table 1), whereas all of them were not different among the vines assigned to the different treatments (Table S1). The shoots of both ‘Aglianico’ and ‘Casavecchia’ had on average 16 main leaves. Conversely, both main and lateral shoots of ‘Casavecchia’ were longer than in ‘Aglianico’ vines (+31% and 45%, respectively). The blade area of the main leaves of ‘Casavecchia’ was 12% smaller than ‘Aglianico’. The internode length was 2 cm longer in ‘Casavecchia’ than in ‘Aglianico’. ‘Casavecchia’ shoots also had a slightly larger number of laterals and of leaves on the laterals than ‘Aglianico’ vines.

**Figure 1:**
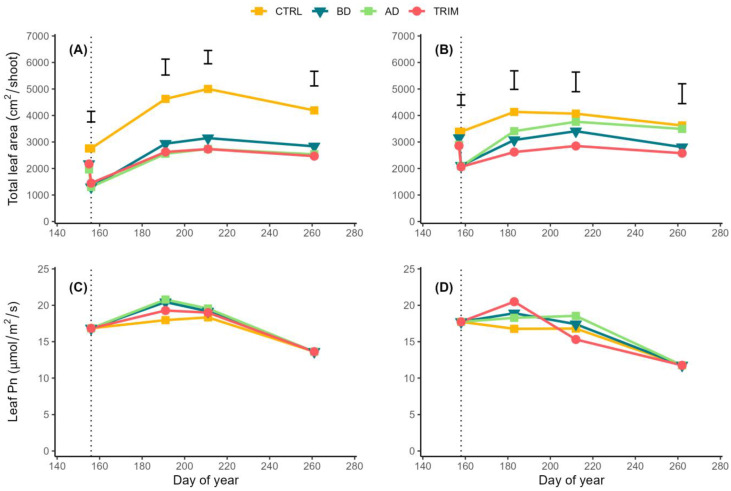
Seasonal pattern of total shoot leaf area (**A**,**B**) and leaf net photosynthetic rate, Pn (**C**,**D**) in ‘Aglianico’ (**A**,**C**) and ‘Casavecchia’ (**B**,**D**) vines exposed to four canopy management treatments (CTRL: control; BD: basal defoliation; AD: apical defoliation; TRIM: shoot trimming). Separately for each measuring data, vertical bars indicate least significant differences (LSD) between treatments (LSDs were calculated only when differences between treatments were significant according to one-way ANOVA). Dotted vertical line indicate the dates of treatment application (day of year 156 and 158 for ‘Aglianico’ and ‘Casavecchia’, respectively).

The three pre-bloom pruning strategies (Figure 1A,B) induced similar levels of total leaf area per shoot (1350 and 2095 cm^2^, respectively, in ‘Aglianico’ and ‘Casavecchia’). This represents an average leaf area reduction of −51% and −38% in ‘Aglianico’ and ‘Casavecchia’, respectively, compared to the initial conditions. In ‘Aglianico’ grapevines, these differences were maintained throughout the duration of vegetative growth. In ‘Casavecchia’, this occurred only for the shoot trimming CM (TRIMM) treatment, whereas the differences in total shoot leaf area between the control CM (CTRL) vines and those exposed to the two defoliation treatments, basal defoliation (BD) and apical defoliation (AD), progressively decreased becoming not significant starting on DOY 211 (55 days after the application of the treatments).

### 2.2. Leaf Net Photosynthetic Rate

**Figure 2:**
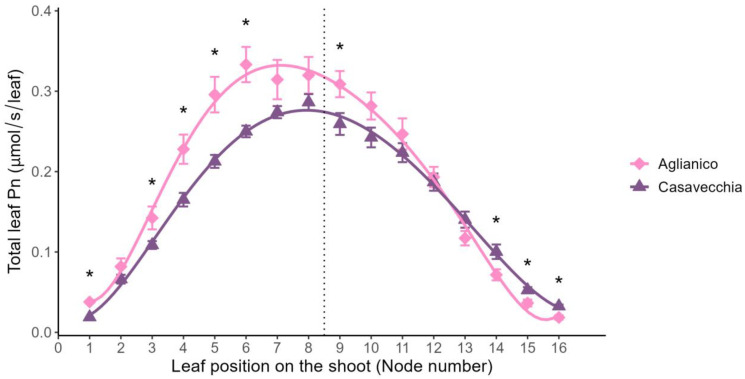
Estimated total net photosynthetic rate of leaves located at different shoot nodes of ‘Aglianico’ and ‘Casavecchia’ grapevines. Error bars are the standard errors of the mean. Asterisks indicate significant differences between cultivars for each leaf position (one-way ANOVA, *p* ≤ 0.05). A dotted vertical line separates the basal leaves (nodes from 1 to 8) removed in vines exposed to basal defoliation from the apical leaves (nodes from 9 to 16) removed in vines exposed to apical defoliation or shoot trimming.

Independently of the node considered, ‘Aglianico’ vines had an estimated total leaf net photosynthesis higher than ‘Casavecchia’ (0.189 ± 0.008 vs. 0.164 ± 0.005 μmol/s/leaf). In both cultivars, the estimated total net photosynthetic rate of the main leaves varied depending on the position (node) along the shoot following a bell-shaped function (Figure 2). This parameter increased from the base of the shoot reaching a relative maximum in the central part of the shoot and then decreased progressively until the apex. The estimated total net photosynthetic rate of leaves located at the 1st node, those between the 3rd and the 6th nodes, and at 9th node was higher in ‘Aglianico’ than ‘Casavecchia’, whereas the opposite occurred for the three most apical leaves (Figure 2).

In both cultivars, net photosynthetic rate per unit leaf area was not affected by the CM ranging between 11.8 and 20.8 μmol/m^2^/s during the growing season (Figure 1C,D). The estimated total shoot photosynthetic rate, measured right after the application of the treatments (stage J), significantly differed between cultivars (29% higher in ‘Casavecchia’) and canopy managements, whereas this parameter was not affected by the interaction between the two factors (Table 2). CM induced on average a 45% decrease in the estimated total shoot photosynthetic rate compared to the control.

### 2.3. Inflorescence Fertility, Fruit Set, and Bunch Architecture at Harvest

‘Casavecchia’ vines had inflorescences with around 30% more flowers than ‘Aglianico’ (Table 2). Vines used for the canopy management trial were homogeneous in terms of inflorescence fertility as suggested by the non-significant effect of CM treatments and the CV × CM interaction on the number of flowers per inflorescence measured before their application. The number of berries per bunch at harvest was not affected by CV (on average of 113 berries/bunch) and CV × CM, whereas CM treatments significantly influenced this parameter (Table 2). Berry number per bunch was increased by shoot trimming compared to vines exposed to both basal and apical (+35%), whereas control vines had intermediate values.

**Figure 3:**
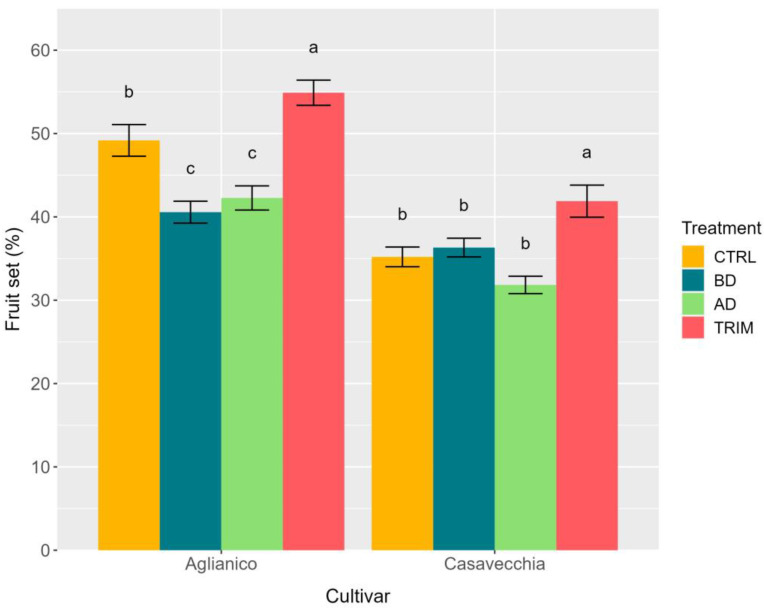
Fruit set in ‘Aglianico’ and ‘Casavecchia’ grapevines exposed to four canopy management treatments (CTRL: control; BD: basal defoliation; AD: apical defoliation; TRIM: shoot trimming). Vertical bars indicate standard errors of the means. Separately for the cultivars, different letters indicate significant differences between canopy management treatments according to the Tukey test (*p* ≤ 0.05).

Fruit set was significantly affected by CV, CM, and CV × CM (Table 2 and Figure 3). Fruit set was significantly higher (around 11%) in ‘Aglianico’ than in ‘Casavecchia’. In both cultivars, shoot trimming induced a significant increase in fruit set compared to control vines (+6% and +7% in ‘Aglianico’ and ‘Casavecchia’, respectively). In ‘Aglianico’, basal and apical defoliation induced a 5% decrease in fruit set, whereas these two CM treatments did not affect it (Figure 3).

Bunch rachis at harvest was more than twice as long in ‘Casavecchia’ than in ‘Aglianico’ vine (Table 2), whereas this morphological trait was not affected by the CM. Independently of the cultivar, berry fresh weight at harvest was significantly decreased in TRIM vines compared to the control and BD vines (Table 2), whereas AD vines had an intermediate berry mass. The CV and CV × CM did not affect this parameter. Bunch compactness indices BCI-1 and BCI-2 were, respectively, 144% and 122% higher in ‘Aglianico’ than in ‘Casavecchia’ (Table 2). Both indices were significantly affected by the CM treatments but not by the CV × CM interaction. These indices were slightly, but significantly, higher in TRIM vines than the other treatments (only than AD vines in the case of IC2).

### 2.4. Bud Potential Fertility, Fruit Yield Components, and Berry Composition at Harvest

The bud potential fertility index was 36% higher in ‘Aglianico’ than in ‘Casavecchia’ vines (Table 3), while it did not differ among vines assigned to the different CM treatments. Similarly, ‘Aglianico’ vines had a significantly higher fruit yield (+69%) and number of bunches per vine (+74%) than ‘Casavecchia’ vines, whereas bunch fresh weight at harvest did not differ between cultivars (an average of 200 g/bunch). Conversely, CM treatments and the CV × CM interaction did not affect fruit yield and the two main yield components (Table 3).

Berry composition at harvest was significantly influenced by the CV and the CM, while the CV × CM interaction did not affect these parameters (Table 3). Berry juices of ‘Aglianico’ grapevines lower TSS (−6%) and pH (−7%), and higher TA (+73%) than ‘Casavecchia’. Independently of the cultivar, apical defoliation induced a slight statistically significant increase in berry juice TSS (+3%) and pH (2%) than the other treatments (in the case of pH, there was no difference between AD and control vines) and decrease in TA (−10%) compared to AD berries.

## 3. Discussion

The alteration of the source–sink relationship is the key target of agronomic strategies to modify bunch compactness [4]. If the limitation of the generalization is accepted, the main source of photoassimilates for berries is the photosynthesis of the leaves, especially those proximal to sink organs. It was previously established that both pre-bloom defoliation and shoot trimming affect fruit set in grapevines [2,17,32]. In this study, we demonstrated that the position of the removed leaves on the shoot (apical or basal) and the canopy management strategy adopted (defoliation or shoot trimming) differentially altered fruit set. Moreover, some of these effects depend also on the genetic factors that underline the different bunch compactness of the two varieties under investigation.

Irrespective of the cultivar, pre-bloom shoot trimming induced a significant increase in fruit set and in the number of berries per bunch, compared to control vines. Shoot trimming and apical defoliation removed the same number of apical leaves of the main shoot. Moreover, these CM practices preserved the same amount of the estimated total shoot photosynthesis (Table 1). Nonetheless, it is noteworthy that only the TRIM CM stimulated fruit set (Table 2). Therefore, this effect underlines the predominant importance of the removal shoot apex in the inhibition of inflorescence along with the cutting of leaves. During early phenological stages of the annual grapevine development, shoot apexes are strong carbon sinks, hence competing with the setting fruits [15,24,33,34]. The comparison between ‘Casavecchia’ and ‘Aglianico’ adds that the role of the shoot apex removal is more relevant for cultivars with a lower fruit set (Table 2). The different response of fruit set to trimming between the two studied cultivars should be also related to differences in the intensity of sink-to-sink (i.e., setting flowers-to-shoot apex) competition. ‘Casavecchia’ has a higher vigor of compared to ‘Aglianico’, as indicated by the longer main shoots (because of longer internodes) and higher number of lateral shoots, traits that are consistent with a higher sink-strength of the vegetative apexes. However, in both cultivars, the increase in fruit set was not associated to a significant increase in fruit yield per vine because a compensatory reduction of fresh weight of the berries occurred in trimmed vines. The very early (in fruit development) increase in the number of berries observed in TRIMM vines may have increased berry-to-berry competition for carbohydrates from the very beginning of the stage I of the berry double-sigmoid growth curve. It is well established that in grapevines, carbon source limitation results in a reduced berry growth, and the sooner these limitations occur, the stronger this effect is [35,36]. The reduction in berry fresh weight found in TRIM ‘Casavecchia’ also has interesting implications for winemakers. Small berries are characterized by a high skin-to-pulp ratio, and winemaking is characterized by an increased anthocyanin extraction from berry skin [37,38].

In both cultivars, shoot trimming resulted in a significant increase in the two bunch compactness indices. Considering the large difference in bunch compactness between the ‘Aglianico’ and ‘Casavecchia’ (mainly due to a rachis with a total length almost double in the latter cultivar), these increases in BCI-1 and BC-2 may not make the difference, in term of susceptibility to bunch rot, for ‘Casavecchia’, whereas they may be strongly undesired in ‘Aglianico’.

The results of this study on the cultivar ‘Aglianico’ confirm that early basal leaf removal is a suitable canopy management practice to decrease the percentage of fruit set in grapevines as previously reported for other varieties [13,15,16,17,39], including ‘Aglianico’ [14]. Interestingly for this cultivar, leaf removal applied to the apical leaves (AD) produced similar effects on fruit set compared to basal defoliation (Figure 2). This result is consistent with the fact that, despite the type of leaf removed (basal or apical), both defoliation strategies resulted in similar levels of the estimated total shoot photosynthetic rate during the phenological stage between anthesis and fruit set (Table 2; Figure 2). This is consistent with other findings on other grapevine cultivars [10,14,16,31], suggesting vines may adjust to carbon starvation in different ways, possibly relying on starch reserves rather than increase photosynthetic rate [40]. Conversely, irrespective of the type of leaf removed, pre-bloom defoliation was ineffective in modulating fruit set in ‘Casavecchia’ grapevines. This suggests that the leaf area retained by the ‘Casavecchia’ vines after the application of AD and BD canopy managements (an average of 2095 cm^2^ vs. 1300 cm^2^ of ‘Aglianico’) did not limit fruit set. Finally, the impact of the BD and TRIM canopy managements on berry composition was not significant, whereas AD induced a slight delay in berry ripening, as suggested by the increase in TA associated to a decrease in TSS in berry juice at harvest compared to the other treatments [41]. To our knowledge, this is the first time that experimental evidence supports apical defoliation as an alternative early CM to basal leaf removal to manipulate fruit set in grapevines.

In conclusion, this work provided a better understanding of the components that are altered by different CM strategies in relation to the bunch compactness. The variation of the main fruit-related factors that determine compaction indicated the physiological interrelation between shoot apexes and fruits, and the possible relevance of the related compensatory mechanisms in terms of supply to the developing inflorescence, although further metabolomics studies should determine likely differences in carbohydrate mobilization. In addition, our work made evident the strong role of the different bunch architecture, and vine vigor of the specific variety to be managed. Specifically, the negligible effect on rachis length for all treatments and cultivars suggest that agricultural management strategies to alter bunch compactness should mainly focus on berries.

Finally, within the proposed management strategies, pre-bloom BD or AD application appears to be suitable CM for controlling compactness in cultivars, such as ‘Aglianico’, characterized by high fruit set, short bunch rachis, and medium-low vigor in early stages of annual cycle, whereas pre-bloom TRIM appears to be a promising CM strategy to enhance fruit set in cultivars, such as ‘Casavecchia’, having relatively low fruit set, long bunch rachis, and high early vigor.

## 4. Materials and Methods

### 4.1. Experimental Site and Plant Material

The trial was conducted in 2008 in a rainfed commercial vineyard located in Galluccio, Caserta, Italy (41°20′45.5″ N 13°56′51.5″ E; 291 m above sea level). The vineyard was planted in 2000 with ‘Aglianico’ and ‘Casavecchia’ grapevines (*Vitis vinifera* L.) grafted onto 1103P rootstock. Vines were trained to a bilateral Guyot leaving, with dormant pruning, two horizontal 1-year-old canes bearing 8 buds each, and two 2-bud spurs per plant. Vine spacing was 2.60 m × 1.50 m (corresponding to a planting density of 2564 vines/ha) and rows had a north-south orientation. The soil was of volcanic origin; according to the USDA Soil Taxonomy [42], the soil was Humic Haploxerands pumiceous, glassy, and thermic. The soil was predominantly sandy (64% sand, 53% silt, 11% clay), and soil depth was around 80 cm. Vineyard was managed following the protocol for the commercial wine production under the denomination of origin mark “Galluccio DOC” (http://www.agricoltura.regione.campania.it/viticoltura/disciplinari/DOC_Galluccio.pdf; accessed on 1 December 2022). This also included a late shoot trimming that was applied to the vines of all treatments on 14 July.

### 4.2. Experimental Design and Treatments

The experimental design was a complete randomized block design with four early (pre-bloom) canopy management treatments, two cultivars (‘Aglianico’ and ‘Casavecchia’), and three blocks. The canopy management treatments were: (a) basal defoliation (BD): 50% of the main leaves was removed starting from the base of the shoot; (b) apical defoliation (AD): 50% of the main leaves was removed starting from the top of the shoot; (c) shoot trimming (TRIM): shoots were trimmed removing 50% of the main leaves; and (d) control (CTRL): no pruning (leaf removal or trimming) was applied in pre-bloom. All the treatments were applied at pre-anthesis, phenological stage H [43], which occurred on 4 and 6 June 2008 (day of year, DOY, 156 and 158, respectively) in ‘Aglianico’ and ‘Casavecchia’ grapevines, respectively. Each treatment was applied to a total of 12 vines per block (corresponding to a total of 36 vines/treatment), but measurements were carried out on the three central vines per block (the other 9 vines per block were used as borders).

### 4.3. Vegetative Growth Measurements

The vegetative measurements were carried out separately on two shoots selected on each of the vine involved in the experiment (a total of 18 shoots per treatment) on five dates per cultivar (‘Aglianico’: 3 and 4 June, 1 and 30 July, and 18 September, corresponding to DOY 155, 156, 183, 212, and 262, respectively; ‘Casavecchia’: 5 and 6 June, 1 and 30 July, and 18 September, corresponding DOY 157, 158, 183, 212 and 262, respectively). On each date, the measured parameters were: length of the main shoot, length of each lateral shoot; number of leaves on each main or lateral shoot, length, and width of the blade of all the leaves located both on the main and the lateral shoots. All these measurements were carried out with a measuring tape. The internode length of the main shoot axis was estimated dividing the shoot length by the number of leaves. In addition, on DOY 156, two samples of 50 leaves (one per cultivar) were separately collected from nearby vines and the length, width, and area of the blade were individually measured in the laboratory (L_leaf_, W_leaf_, A_leaf_, respectively). The latter parameter was measured with a leaf area meter (LI-3100, LI-COR, Inc., Lincoln, NB, USA). The cultivar-specific linear relationships between A_leaf_ and the product L_leaf_ × W_leaf_ (‘Aglianico’: A_leaf_ = 1.01542 × (L_leaf_ × W_leaf_), R^2^ = 0.97, *p* < 0.001; ‘Casavecchia’: A_leaf_ = 0.83055 × (L_leaf_ × W_leaf_), R^2^ = 0.97, *p* < 0.001) were used to estimate the blade area of each leaf of the selected shoots using the measured L_leaf_ and W_leaf_ as inputs.

### 4.4. Leaf Photosynthetic Rate Measurements

Leaf photosynthetic rate per unit leaf area was measured on four dates per cultivar (‘Aglianico’: 4 June, 9 and 29 July, and 17 September, corresponding to DOY 156, 191, 211, and 261, respectively; ‘Casavecchia’: 6 June, 1 and 30 July, and 18 September 2008, corresponding to DOY 158, 183, 212, and 262, respectively). On each date, measurements were carried out with a portable gas exchange analyzer (LCA 4, ADC BioScientific Ltd., Hoddesdon, UK) within two hours around solar noon (11:00–13:00) under saturating PAR conditions (>1900 μmol/m^2^/s). On the first date (DOY 156 and 158 for ‘Aglianico’ and ‘Casavecchia’, respectively), the measures were taken, right before the canopy management treatments on eight leaves located on every other node of the two previously selected shoots on three vines per cultivar (a total of 6 shoots/cultivar). The photosynthetic rate per unit leaf area of each of the unmeasured leaf was assumed to be equal to the mean of the photosynthetic rates of the two adjacent leaves (located on the previous and the following node). The total photosynthetic rate of each leaf on the shoot was estimated, multiplying its photosynthetic rate per unit area by its blade area. On the other three measuring dates, for each treatment and cultivar, the photosynthetic rate was measured on nine (three per block) fully developed and well-exposed main leaves located in the middle part of the shoot. The total shoot photosynthetic rate was estimated, multiplying photosynthetic rate per unit leaf area by the whole shoot leaf area.

### 4.5. Vine Fertility

All the bunches located on the two selected shoots were individually photographed with a digital camera at the phenological stages H [43] that occurred on 4 and 6 June for ‘Aglianico’ and ‘Casavecchia’ grapevines, respectively. The number of flowers visible in the pictures (NF_pic_) was counted. On the same dates (stage H), 30 bunches located on nearby vines were photographed and then collected to count both the NF_pic_ and the total real number of flowers located on them (real number of flowers per bunch, NF_real_ using previously described procedures [10]. In addition, at phenological stage J [43], the number of bunches on each shoot of the selected vines was separately counted and averaged to calculate the bud potential fertility index.

### 4.6. Fruit Yield Components, Fruit Set, and Berry Composition at Harvest

Harvest was carried out when berry juice reached a total soluble content (TSS) of 21 °Brix. (2 and 20 October 2008 for ‘Casavecchia’ and ‘Aglianico’, corresponding to DOY 276 and 294, respectively). To decide harvest time, TSS was monitored every 5–7 days (starting 15 days before the expected date) on a sample of 75 berries/treatment (5 berries/vine) with a digital refractometer (RFM81, Bellingham + Stanley, Kent, UK). At harvest, the bunches located on each vine were separately counted and weighed. These data were used to calculated mean bunch weight. The bunches used for counting the number of flowers (at stage H) were transported to the laboratory to individually count the number of berries per bunch and the total length of the rachis (main axis plus wings). Two bunch compactness indices were calculated, respectively, as the ratio between berry number and total rachis length (BCI-1) and as the ratio between bunch weight and total rachis length (BCI-2) [4].

In addition, samples of 20 berries per vine (a total of 180 berries/treatment) were collected for all treatments to determine berry composition at harvest. TSS was measured as previously described, whereas titratable acidity was measured by adding 1N NaOH until pH 8.2. During titration pH was monitored continuously with a digital pHmeter (GLP 21, Crison, Alella, Barcelona, Spain).

### 4.7. Meteorological Data

Air temperature and rainfall were measured hourly at a nearby weather station (41°21′27.5″ N 14°6′7.8″ E). In 2008, total annual rainfall was 1088 mm, and the average the average growing-season temperature (between 1 April and 31 October; [44]) as 19.3 °C. The seasonal patterns of daily minimum, maximum, and mean air temperature and of daily rainfall are reported in Supplementary Figure S1.

### 4.8. Statistical Analysis

The significance of the effect of the cultivar (CV), the canopy management (CM), and the CV × CM interaction on the different parameters measured after the application of the CM treatments was assessed by two-way ANOVA using the Tukey’s honestly significant difference (HSD) test for mean separation (*p* ≤ 0.05). The significance of the difference between cultivars in the estimated total leaf net photosynthetic rate at the different shoot nodes and in all the vegetative parameters measured before the application of the CM treatments was assessed by one-way ANOVA (*p* ≤ 0.05). To help visually highlight the significance differences between treatments in total shoot leaf area and photosynthetic rate, the least significant differences (LSD) were calculated and reported in Figure 1. All the statistical analyses and graphs were done with R 4.2 [45].

## Figures and Tables

**Table 1:** Difference between ‘Aglianico’ and ‘Casavecchia’ vines in shoot vegetative characteristics measured at the phenological stage H, pre-anthesis (just before the application of the experimental treatments). N.s., *, and ***: Not significant, or significant at *p* ≤ 0.05, and 0.001, respectively.

Source of Variation	Main Shoot Length (cm)	Number of Main Leaves on the Shoot	Internode Length of the Main Shoot (cm)	Area of the Main Leaves (cm^2^/leaf)	Number of Lateral Shoots on the Main Shoot	Lateral Shoot Length (cm/lateral)	Number of Leaves per Lateral
Cultivar (CV)							
‘Aglianico’	100.0 ± 2.2	16.4 ± 0.2	6.1 ± 0.1	110.8 ± 2.9	7.4 ± 0.3	3.8 ± 0.1	2.2 ± 0.1
‘Casavecchia’	131.2 ± 3.2	16.0 ± 0.2	8.2 ± 0.2	97.0 ± 2.0	8.9 ± 0.3	5.5 ± 0.1	2.5 ± 0.1
Significance	***	n.s.	***	***	***	***	*

**Table 2:** Effect of the cultivar (CV: ‘Aglianico’ and ‘Casavecchia’), canopy management (CM: control, basal defoliation, apical defoliation, shoot trimming) and the CV × CM interaction (assessed by two-way ANOVA) on the number of flowers per bunch (at stage H before the application of the CM treatments), estimated total shoot photosynthetic rate measured at stage H right after the application of canopy management treatments. and number of berries per bunch, fruit set total rachis length, berry fresh weight, and the two bunch compactness indices (BCI-1 and BCI-2) measured at harvest. N.s., *, **, and ***: not significant, or significant at *p* ≤ 0.05, 0.01, and 0.001, respectively. Separately for each source of variation and within each column, means followed by different letters are significantly different according to the Tukey test (*p* ≤ 0.05).

Source of Variation	No Flowers/INFLORESCENCE	Tot Pn per Shoot (Main + Laterals) after trt (μmol/s/shoot)	No Berries/Bunch	Fruit Set (%)	Total Rachis Length (cm)	Berry Fresh Weight (g/berry)	BCI-1 (No berries/cm)	BCI-2 (g berries/cm)
Cultivar (CV)								
‘Aglianico’	239 ± 9 b	2.80 ± 0.17 b	114 ± 5 a	47.0 ± 0.9 a	36.9 ± 1.6 b	1.86 ± 0.04 a	3.26 ± 0.12 a	5.50 ± 0.18 a
‘Casavecchia’	309 ± 15 a	3.61 ± 0.2 a	112 ± 6 a	36.1 ± 0.8 b	84.1 ± 3.8 a	1.90 ± 0.03 a	1.34 ± 0.04 b	2.48 ± 0.09 b
Significance	***	***	n.s.	***	***	n.s.	***	***
Canopy management (CM)								
Control	274 ± 21 a	4.8 ± 0.29 a	119 ± 10 ab	42.9 ± 1.6 b	56.5 ± 4.9 a	1.96 ± 0.07 a	2.35 ± 0.12 b	4.17 ± 0.29 ab
Basal Defoliation	270 ± 19 a	2.64 ± 0.2 b	103 ± 7 b	38.7 ± 0.9 c	59.4 ± 6.3 a	1.96 ± 0.07 a	2.28 ± 0.19 b	4.31 ± 0.35 ab
Apical Defoliation	259 ± 13 a	2.62 ± 0.18 b	96 ± 4 b	37.6 ± 1.2 c	55.3 ± 4.2 a	1.89 ± 0.05 ab	2.10 ± 0.14 b	3.65 ± 0.24 b
Shoot Trimming	272 ± 14 a	2.74 ± 0.24 b	135 ± 8 a	49.8 ± 1.5 a	57.9 ± 4.8 a	1.72 ± 0.03 b	2.99 ± 0.25 a	4.68 ± 0.32 a
Significance	n.s.	***	***	***	n.s.	**	***	*
CV × CM								
Significance	n.s.	n.s.	n.s.	**	n.s.	n.s.	n.s.	n.s.

**Table 3:** Effect of the cultivar (CV: ‘Aglianico’ and ‘Casavecchia’), canopy management (CM: control, basal defoliation, apical defoliation, shoot trimming) and the CV × CM interaction (assessed by two-way ANOVA) on bud potential fertility index, fruit yield, number of bunches per vine, bunch fresh weight, and berry juice total soluble solids, pH, and titratable acidity at harvest. N.s., *, **, and ***: not significant, or significant at *p* ≤ 0.05, 0.01, and 0.001, respectively. Separately for each source of variation and within each column, means followed by different letters are significantly different according to the Tukey test (*p* ≤ 0.05).

Source of Variation	Bud Potential Fertility Index (No bunches/bud)	Fruit Yield (kg/vine)	No Bunches per Vine	Bunch Fresh Weight (g/bunch)	Total Soluble Solids (°Brix)	pH	Titratable Acidity (% Tartaric Acid)
Cultivar (CV)							
‘Aglianico’	1.64 ± 0.06 a	6.47 ± 0.40 a	33 ± 1 a	194.7 ± 9.3 a	21.3 ± 0.1 b	3.23 ± 0.01 b	0.77 ± 0.01 a
‘Casavecchia’	1.21 ± 0.05 b	3.83 ± 0.25 b	19 ± 1 b	205.4 ± 9.3 a	22.6 ± 0.1 a	3.48 ± 0.01 a	0.45 ± 0.01 b
Significance	***	***	***	n.s.	***	***	***
Canopy management (CM)							
Control	1.36 ± 0.09 a	5.35 ± 0.69 a	24 ± 3 a	222.7 ± 18.2 a	21.8 ± 0.2 b	3.37 ± 0.03 ab	0.62 ± 0.03 ab
Basal Defoliation	1.47 ± 0.10 a	5.21 ± 0.55 a	26 ± 2 a	197.8 ± 11.2 a	21.8 ± 0.2 b	3.33 ± 0.02 b	0.60 ± 0.03 ab
Apical Defoliation	1.42 ± 0.10 a	4.69 ± 0.40 a	27 ± 2 a	179.3 ± 9.7 a	22.4 ± 0.2 a	3.40 ± 0.03 a	0.57 ± 0.02 b
Shoot Trimming	1.44 ± 0.09 a	5.35 ± 0.61 a	26 ± 2 a	200.3 ± 10.4 a	21.7 ± 0.2 b	3.32 ± 0.02 b	0.64 ± 0.03 a
Significance	n.s.	n.s.	n.s.	n.s.	**	**	*
CV × CM							
Significance	n.s.	n.s.	n.s.	n.s.	n.s.	n.s.	n.s.

## Data Availability

The data not already included in the tables, figures, and supporting material of this article will be made available from the corresponding author (B.B.) upon reasonable request.

## Supplementary Materials

The following supporting information can be downloaded at: https://www.mdpi.com/article/10.3390/plants12040733/s1, Table S1: Shoot vegetative characteristics of the vines assigned to the different canopy management (CM) treatments measured at the phenological stage H, pre-anthesis (just before the application of the experimental treatments). The significance of the differences was assessed by one-way ANOVA (*p* ≤ 0.05). Figure S1. Seasonal pattern of daily minimum (azure line), maximum (red line), and mean air temperature (yellow line), and of daily rainfall (blue bars) measured in 2008.
