# Supplementary material for: Early Canopy Management Practices Differentially Modulate Fruit Set, Fruit Yield, and Berry Composition at Harvest Depending on the Grapevine Cultivar
Source: Plants (Basel). 2023 Feb 7;12(4):733. doi: 10.3390/plants12040733 (PMC9959345; doi:10.3390/plants12040733)
Supplement: Supplementary file 1 [file plants-12-00733-s001.zip › Supplementary_Figure S1.pdf]

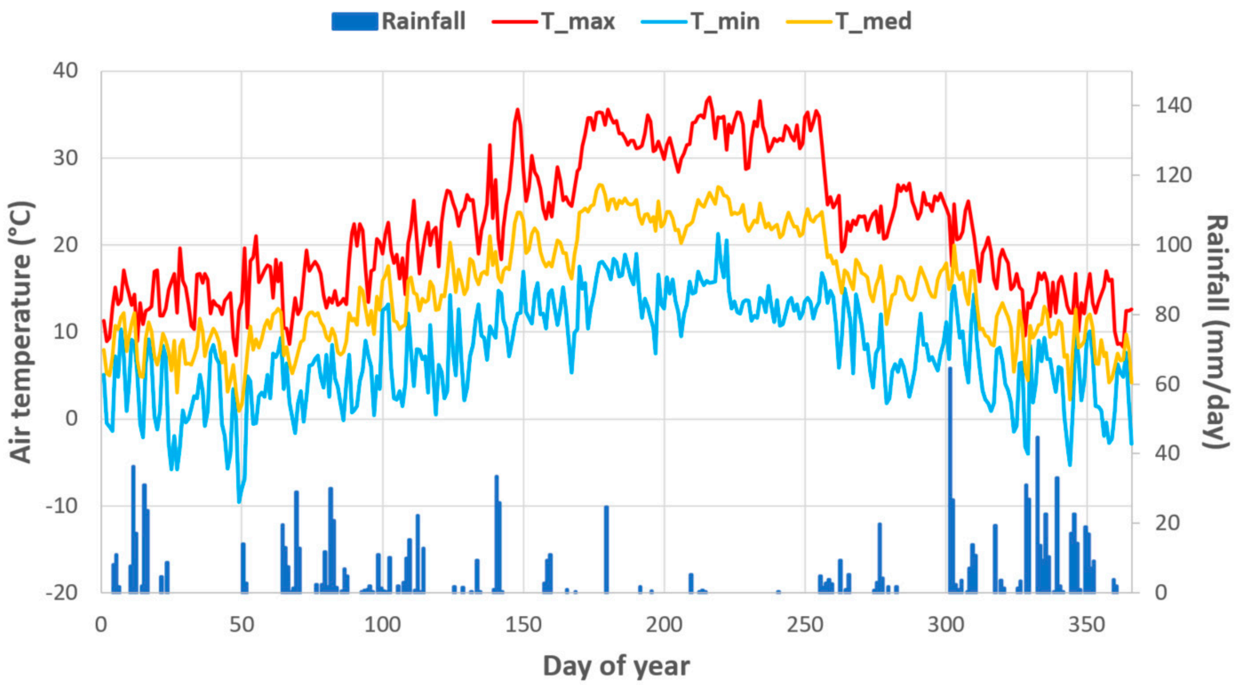

Figure S1. Seasonal pattern of daily minimum (azure line), maximum (red line), and mean air temperature (yellow line), and of daily rainfall (blue bars) measured in 2008.
