# Supplementary material for: Early Canopy Management Practices Differentially Modulate Fruit Set, Fruit Yield, and Berry Composition at Harvest Depending on the Grapevine Cultivar
Source: Plants (Basel). 2023 Feb 7;12(4):733. doi: 10.3390/plants12040733 (PMC9959345; doi:10.3390/plants12040733)
Supplement: Supplementary file 1 [file Supplementary_Table S1.pdf › Supplementary_Table S1.pdf]

Table S1. Shoot vegetative characteristics of the vines assigned to the different canopy management (CM) treatments measured at the phenological stage H, pre-anthesis (just before the application of the experimental treatments). The significance of the differences was assessed by one-way ANOVA ($p \leq 0.05$).

Source of Variation	Main shoot length (cm)	Number of main leaves on the shoot	Internode length of the main shoot (cm)	Area of the main leaves (cm^2/leaf)	Number of lateral shoots on the main shoot	Lateral shoot length (cm/lateral)	Number of leaves per lateral
Canopy management (CM)							
Control	122.2±5.0a	16.4±0.2a	7.5±0.3a	111.2±4.3a	8.9±0.4a	4.6±0.2a	2.7±0.15a
Basal Defoliation	115.0±4.6a	16.4±0.3a	7.0±0.3a	103.4±3.0a	8.1±0.5a	4.6±0.2a	2.4±0.1a
Apical Defoliation	116.1±4.5a	16.0±0.3a	7.2±0.3a	99.1±3.2a	8.0±0.4a	4.6±0.2a	2.1±0.1a
Shoot Trimming	109.2±4.5a	16.1±0.3a	6.8±0.3a	101.8±4.0a	7.7±0.4a	4.9±0.2a	2.2±0.1a
Significance	n.s.	n.s.	n.s.	n.s.	n.s.	n.s.	n.s.
